# Biallelic *BUB1* mutations cause microcephaly, developmental delay, and variable effects on cohesion and chromosome segregation

**DOI:** 10.1126/sciadv.abk0114

**Published:** 2022-01-19

**Authors:** Sara Carvalhal, Ingrid Bader, Martin A. Rooimans, Anneke B. Oostra, Jesper A. Balk, René G. Feichtinger, Christine Beichler, Michael R. Speicher, Johanna M. van Hagen, Quinten Waisfisz, Mieke van Haelst, Martijn Bruijn, Alexandra Tavares, Johannes A. Mayr, Rob M. F. Wolthuis, Raquel A. Oliveira, Job de Lange

**Affiliations:** 1 Instituto Gulbenkian de Ciência, R. Q.ta Grande 6, 2780-156 Oeiras, Portugal.; 2 Algarve Biomedical Center Research Institute, Universidade do Algarve, 8005-139 Faro, Portugal.; 3 Centre for Biomedical Research, Universidade do Algarve, 8005-139 Faro, Portugal.; 4 Unit of Clinical Genetics, Paracelsus Medical University, Salzburg, Austria.; 5 Cancer Center Amsterdam, Amsterdam University Medical Centers, Oncogenetics Section, De Boelelaan 1118, 1081 HV Amsterdam, Netherlands.; 6 Department of Pediatrics, University Hospital Salzburg, Paracelsus Medical University, Salzburg, Austria.; 7 Institute of Human Genetics, Diagnostic and Research Center for Molecular BioMedicine, Medical University of Graz, Graz, Austria.; 8 Department of Clinical Genetics, Amsterdam UMC, Vrije Universiteit Amsterdam, De Boelelaan 1118, 1081 HV Amsterdam, Netherlands.; 9 Northwest Clinics, Wilhelminalaan 12, 1815 JD Alkmaar, Netherlands.

## Abstract

Budding uninhibited by benzimidazoles (BUB1) contributes to multiple mitotic processes. Here, we describe the first two patients with biallelic *BUB1* germline mutations, who both display microcephaly, intellectual disability, and several patient-specific features. The identified mutations cause variable degrees of reduced total protein level and kinase activity, leading to distinct mitotic defects. Both patients’ cells show prolonged mitosis duration, chromosome segregation errors, and an overall functional spindle assembly checkpoint. However, while BUB1 levels mostly affect BUBR1 kinetochore recruitment, impaired kinase activity prohibits centromeric recruitment of Aurora B, SGO1, and TOP2A, correlating with anaphase bridges, aneuploidy, and defective sister chromatid cohesion. We do not observe accelerated cohesion fatigue. We hypothesize that unresolved DNA catenanes increase cohesion strength, with concomitant increase in anaphase bridges. In conclusion, *BUB1* mutations cause a neurodevelopmental disorder, with clinical and cellular phenotypes that partially resemble previously described syndromes, including autosomal recessive primary microcephaly, mosaic variegated aneuploidy, and cohesinopathies.

## Teaser

Biallelic germline mutations in a gene important for mitotic fidelity, BUB1, are first described and functionally assessed.

## INTRODUCTION

Chromosome segregation is a tightly organized process that is essential for maintaining genomic stability. Following DNA condensation and nuclear envelope breakdown (NEB), duplicated sister chromatids interact with the mitotic spindle, which consists of microtubules emanating from both polar centrosomes. These interactions enable chromosome alignment and biorientation at the spindle equator. The kinetochore, a large protein complex assembled at the centromere, serves as the microtubule attachment site and facilitates chromosome movement in mitosis. Chromosome biorientation is further supported by the protein complex cohesin, which tethers sister chromatids together to resist pulling forces generated by the microtubules. Incorrect kinetochore-microtubule attachments are destabilized and eliminated, allowing a new attachment round. During this alignment process, unattached kinetochores activate the spindle assembly checkpoint (SAC), a signaling pathway that inhibits progression to anaphase. When all chromosomes are correctly connected to the mitotic spindle, the SAC is inactivated, and its downstream target, the anaphase-promoting complex/cyclosome (APC/C), is released. Subsequently, cohesin is cleaved in a rapid, protease-dependent fashion, allowing the irreversible separation of sister chromatids to opposite poles of the cell [for reviews, see (*1*, *2*)].

Mitotic defects can lead to chromosome missegregation, potentially resulting in the loss or gain of whole chromosomes, known as aneuploidy. Aneuploidy is generally detrimental for proliferation. At the same time, however, aneuploidy is a cancer hallmark, and chromosomal instability (CIN; an elevated rate of chromosome missegregation) is considered to be a driving force in the genesis and evolution of human cancers (*3*). A wide range of clinically diverse genetic diseases are caused by mutations in mitosis-associated genes. These include, for example, defects in centriole biogenesis, kinetochore components, and sister chromatid cohesion (*4*–*7*). A common characteristic of several syndromes associated with mitotic genes is microcephaly, possibly related to abnormal neuronal cell proliferation or elevated levels of neuronal stem cell death (*4*, *5*). Primary microcephaly (MCPH) is associated with a broad spectrum of other clinical traits where 22 of 25 known genes are linked with mitotic functions (*5*). Mosaic variegated aneuploidy (MVA), caused by biallelic mutations in genes encoding the kinetochore proteins BUBR1, CEP57, TRIP13, or CENATAC, often predisposes to early childhood cancer and displays aneuploidies of random chromosomes in >5% of cells in different tissues (*8*–*12*). The cohesinopathies Roberts syndrome (RBS), Warsaw breakage syndrome (WABS), and chronic atrial and intestinal dysrhythmia (CAID) are characterized by cohesion loss and/or spontaneous railroad chromosomes (*13*–*15*). No clear cancer predisposition is found in patients with cohesinopathy (*7*). Notably, aneuploidy is occasionally observed in RBS (*16*, *17*), and some patients with MVA exhibit premature chromatid separation (PCS) (*10*).

Budding uninhibited by benzimidazoles (BUB1) is a multifunctional component of the segregation machinery. The protein contains an N-terminal kinetochore localization domain, multiple binding motifs, and a C-terminal kinase domain, enabling both kinase-dependent and kinase-independent activities (*18*). BUB1 stably binds unattached kinetochores and acts as a scaffold for the assembly of the mitotic checkpoint complex (MCC), composed of BUBR1, BUB3, MAD2, and CDC20, which restricts APC/C activity (*19*, *20*). The importance of BUB1 for SAC function appears to be context-dependent. In human cells, BUB1-depleted cells can still display a functional SAC, unless the SAC is already compromised, indicating that although BUB1 contributes to full SAC activity, it is not absolutely essential (*21*–*24*). BUB1 is also directly involved in chromosome alignment (*25*), in part by preserving a BUBR1 kinetochore pool (*26*). Proper kinetochore-microtubule attachments are further supported by BUB1-dependent centromeric H2A-T120 phosphorylation, which promotes the recruitment of the chromosomal passenger complex, consisting of Aurora B, inner centromere protein (INCENP), survivin, and borealin. In addition, pH2A-T120 promotes the recruitment of Shugoshin 1 (SGO1) and protein phosphatase 2A, thereby protecting centromeric cohesion (*27*–*31*). Last, centromere recruitment of TOP2A was recently shown to depend on BUB1-mediated pH2A-T120 (*32*). TOP2A removes DNA entanglements that arise naturally during DNA replication, thereby preventing DNA breakage during sister chromatid separation (*33*).

Complete disruption of the *BUB1* open reading frame in human cell lines by CRISPR-Cas9 was challenging due to the expression of alternatively spliced variants and was only successful upon deletion of the entire gene in HAP1 cells (*22*–*24*, *34*, *35*). Homozygous *Bub1* disruption in mice is lethal shortly after embryonic day 3.5 (*36*). A hypomorphic *Bub1* mutant mouse [which lacks exons 2 and 3 and expresses <5% of wild-type (WT) protein levels] is viable but exhibits increased tumorigenesis with aging and aneuploidy (*37*). By contrast, a kinase-dead *Bub1* mutant mouse does not show enhanced spontaneous or carcinogen-induced tumorigenesis despite a high frequency of aneuploid cells (*38*). In humans, reduced BUB1 expression is associated with spontaneous miscarriages (*39*). Heterozygous germline *BUB1* mutations are described in a small number of patients with heritable colorectal cancer (CRC), who exhibit reduced expression levels and variegated aneuploidy in multiple tissues (*40*). However, it is debatable whether BUB1 is a CRC predisposition gene (*41*, *42*).

Here, we describe the first two patients with biallelic germline *BUB1* mutations. Both display microcephaly, intellectual disability, developmental delay, and a few patient-specific phenotypic abnormalities. We observe multiple mitotic defects that are partially different between the patients’ cells, likely reflecting differences in the level and activity of their respective residual protein products. Together, biallelic germline *BUB1* mutations underlie a developmental syndrome with characteristics of MCPH, MVA, and cohesinopathies.

## RESULTS

### Identification of two patients with biallelic mutations in *BUB1*

Two unrelated patients with biallelic *BUB1* mutations were recruited via GeneMatcher (*43*). Their main characteristics include growth retardation, microcephaly, and intellectual disability, as well as a few phenotypic abnormalities specific for each patient (Table 1).

**Table 1.** Clinical features of patients. ASD, atrial septal defect; IUGR, intrauterine growth retardation; SGA, small for gestational age; OFC, occipito-frontal circumference; PCC, premature chromosome condensation; cMRI, cranial magnetic resonance imaging, ID, intellectual disability; IQ, intelligence quotient; SNP, single-nucleotide polymorphism; CGH, comparative genomic hybridization; MLPA, multiplex ligation-dependent probe amplification.

	**P1**	**P2**
*BUB1* variants: NM_004336.4	c.[2T>G];[2T>G]	c.[2625+1G>A] mat;[c.2197dupG] pat
Protein	p.[?];[?]	p.[V822_L875del];[D732fs*11]
Zygosity	Homozygous	Compound heterozygous
Mutation detected by	Exome	Exome
Gender	Male	Female
Age at last assessment	3 years 10 months	16 years 10 months
Prenatal	Enlarged nuchal translucency, IUGR, Pierre-Robin sequence	No complications
**Birth**		
Delivery	Normal	Sec. caesarean section due to fetal distress
Gestational week	39 + 4	38 + 5
Length in cm (SD*)	45 (−3)	46 (−2,8)
Weight in g (centiles†)	2400 (0.4th–2nd)	2825 (9th–25th)
OFC in cm (SD*)	32 (−2,8)	31 (−2,9)
**Growth**		
Stature	SGA, short stature	Slight growth retardation
Length in cm (SD*)	89 (−3) under growth hormone substitution since age 3 years 4 months	152,8 (−2)
Weight in kg (centiles)	11 (<0.4th)	74.5 (91st–99th)
OFC in cm (SD*)	41.5 (−7 SD)	49.5 (−4,9)
**Head and neck**		
OFC	Microcephaly	Microcephaly
Ear	Asymmetric ears, uplifted earlobes	No abnormality
Eye	Epicanthic folds, long palpebral fissure	Axenfeld Rieger anomaly both eyes, also present in father (posterior embryotoxon–anterior synechiae)
Face	Pierre-Robin sequence	No abnormality
Mouth	Clefting of soft palate, thin upper lip, small mouth, difficulties with chewing	No abnormality
**Cardiovascular**	Small ASDII	Small ASDII
**Respiratory**	Long tracheal stenosis, tracheostoma	Bronchial obstruction after viral infection
**Genitourinary**	Hypospadia	No abnormality
**Skeletal**		
Scull	Choanalstenosis	No abnormality
Hands	Clinodactyly fifth finger	No abnormality
Feet	Sickle feet as baby, sandal gaps	Broad feet, sandal gaps
**Skin, nails, and hair**		
Skin	No abnormality	Acanthosis nigricans neck, two cafe au lait spots
Hair/teeth	No abnormality	Small teeth, remarkably light hair color compared to parents
**Neurologic**		
Brain imaging	cMRI at age 1 year: symmetrical supratentorial cortical atrophy, enlarged ventricles	Not available
Developmental delay/ID	Yes	IQ 68, mild intellectual disability
Age of walking	1 year 10 months	18 months
First words/current speech	3 active words at the age of 4 years, communication with gestures	Started talking “late,” had speech therapy. With help, she is able to write a few simple words, but reading is difficult (she does not understand what she reads).
Behavior	Happy and friendly	Normal, quiet mental state
**Endocrine feature**	Growth hormone deficiency, supplemented since age 3 years and 4 months	Obesity
**Other**		Hernia inguinalis
**Normal laboratory findings**	Normal results for: invasive prenatal diagnostics (SNP array) and postnatal array CGH from blood	Normal results for: MLPA telomeres; array CGH; SNP array; PCC; metabolic investigations in urine and blood and DNA analysis of PITX2, GREM1, FOXD3, and FOXC1

*Height and head circumference charts: primary microcephaly and primordial dwarfism.

†Centiles from the child growth foundation 1996/2001.

Patient 1 (P1) is a male, second child of reportedly nonconsanguineous parents of Austrian descent. Invasive prenatal diagnostics was performed because of enlarged nuchal translucency, Pierre-Robin sequence, and intrauterine growth retardation of the fetus. At delivery, the patient presented with microcephaly, clefting of the soft palate, choanalstenosis, a small congenital heart anomaly, hypospadia, sickle feet, and a long tracheal stenosis requiring tracheotomy and subsequent surgery. Additional dysmorphic features included uplifted earlobes, thin upper lip, and clinodactyly. Cranial magnetic resonance imaging showed enlarged ventricles and supratentorial cortical atrophy. He has intellectual deficit, and his motor and speech development are delayed. He has short stature and growth hormone deficiency. His behavior is normal with a happy disposition.

P2 is a female, second child of nonconsanguineous parents of Turkish descent with Bulgarian ancestry. She was born after an uncomplicated pregnancy by secondary caesarean section because of fetal distress. At birth, she was microcephalic. She has Axenfeld Rieger anomaly (which is also present in the father and thus seemingly unrelated to the genotype) and a small congenital heart anomaly. The girl has delayed developmental milestones. She has no dysmorphic facial features but has minor phenotypic abnormalities (see Table 1). As an adolescent, she presents with short stature, obesity, microcephaly, mild intellectual disability, and normal, rather quiet, behavior.

**Fig. 1.**
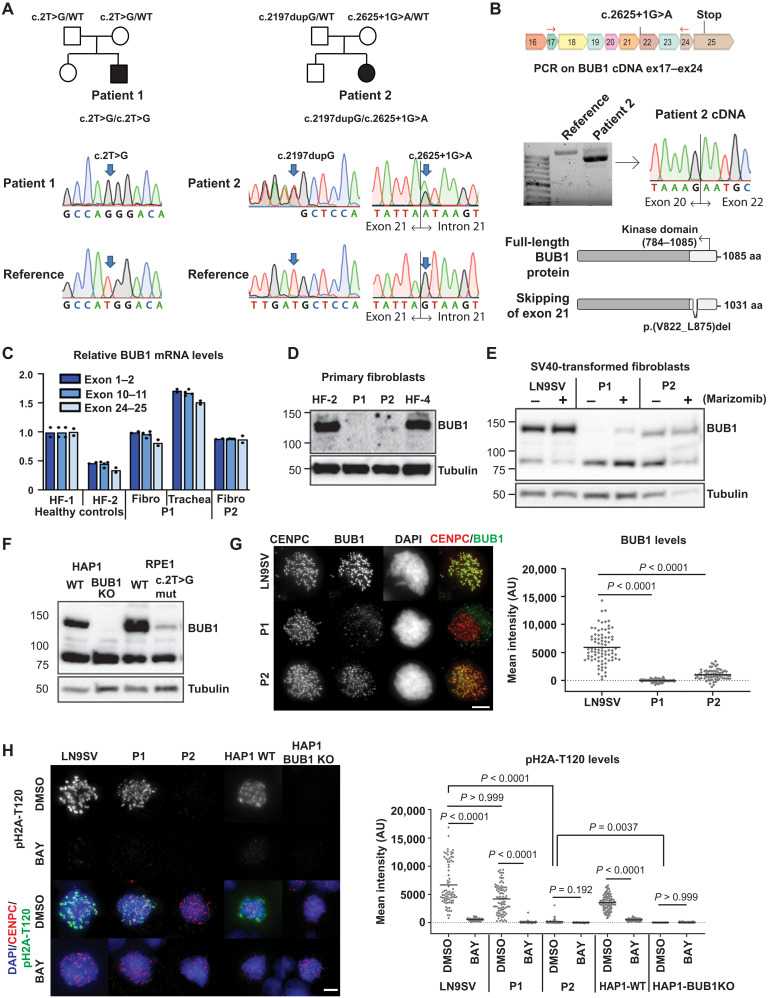
Biallelic germline mutations in BUB1 cause reduced protein levels. (**A**) Family pedigrees (based on WES) and Sanger confirmation from primary fibroblasts. Nomenclature NM_004336.5. (**B**) P2 primary fibroblast cDNA was PCR-amplified using indicated primers (red arrows) and analyzed on agarose gel and by Sanger sequencing. The transcript lacks exon 21. Predicted effect on BUB1 protein is shown. (**C**) BUB1 mRNA was assessed in indicated cells using three different primer pairs, each in three technical replicates. (**D** and **E**) Western blot of primary fibroblasts (D) (using A300-386A to detect BUB1) and SV40-transformed fibroblasts (E) (using ab9000). CDC6 is a control for marizomib-induced protein stabilization (6 hours, 500 nM). (**F**) Western blot of engineered RPE1-BUB1 mutant cells and complete BUB1 KO HAP1 cells using ab9000. (**G**) Colchicine-arrested cells (10 μM, 2 hours) were stained for CENPC, BUB1 (using A300-386A), and DNA. Graph depicts mean BUB1 intensity within CENPC-defined regions. *n* = 83, 73, and 72 cells from three independent experiments. (**H**) Colchicine-arrested cells, treated with BUB1 kinase inhibitor BAY1816032 (4 μM, 1 hour) or dimethyl sulfoxide (DMSO), were stained, and mean pH2A-T120 intensity was assessed within DAPI-defined regions. Approximately 90 cells from three independent experiments were analyzed per condition. Each dot represents one cell; black lines indicate the means. Scale bars, 5 μm. Nonparametric Kruskal-Wallis test (Dunn’s multiple comparison test) used for statistical analysis. AU, arbitrary units; aa, amino acid.

Whole-exome sequencing (WES) revealed biallelic *BUB1* mutations in both patients, which was confirmed by Sanger sequencing (Fig. 1A). P1 harbors a homozygous point mutation in the start codon (c.2T>G) located within a 14-Mb region of homozygosity. Heterozygous carriership of the variant was confirmed by Sanger sequencing of DNA of blood from the parents. The allele frequency in gnomAD is 0.00000854 with no homozygous individuals. P2 contains a splice site mutation in the maternal allele (c.2625+1G>A), which has an allele frequency of 0.00000406 with no homozygous individuals. The paternal allele contains a duplication (c.2197dupG), which is absent in gnomAD. Complementary DNA (cDNA) sequencing revealed that c.2625+1G>A mutation leads to the skipping of exon 21, which results in a transcript with an in-frame deletion of 54 amino acids in the kinase domain of the BUB1 protein (Fig. 1B).

Both patients showed normal BUB1 mRNA levels (Fig. 1C) but severely reduced BUB1 protein levels (Fig. 1D). In line with the cDNA analysis, P2 showed a shorter protein product, corresponding with the predicted kinase deletion mutant. Low levels of full-length BUB1 protein were detectable in SV40-transformed P1 cells, particularly upon treatment with the proteasome inhibitor marizomib (Fig. 1E and fig. S1). To further investigate the effect of the start codon mutation in P1 cells, we edited both *BUB1* alleles of RPE1 cells using CRISPR (Fig. 1F and fig. S1). We used TP53KO cells to enable efficient gene editing, and no additional silent mutations in the HDR template were included. We obtained one pure *BUB1* mutant clone, which indeed showed a small amount of full-length BUB1 protein (Fig. 1F), whereas complete BUB1 knockout (KO) HAP1 cells (*22*) showed no expression. Note that no shorter BUB1 versions could be detected in P1 or mutant RPE1 cells using multiple antibodies (fig. S1), indicating that these cells do not use an alternative start codon to produce an N-terminally truncated protein. In agreement with the Western blot analyses, immunofluorescence of colchicine-treated cells showed that BUB1 recruitment to kinetochores was nearly undetectable in P1 cells, whereas it was reduced in P2 cells (Fig. 1G and fig. S2A), relative to LN9SV, used as a standard control (fig. S2B).

We then set out to investigate to what extent the kinase activity of BUB1 is affected. For this, we measured the phosphorylation of H2A-T120, a major substrate of BUB1 kinase (fig. S2C). P1 cells showed a partial reduction in overall pH2A-T120, with signals appearing more confined to the centromere-proximal region (Fig. 1H and fig. S2D). Treating P1 cells with the BUB1 kinase inhibitor BAY1816032 further decreased pH2A-T120 levels (Fig. 1H), confirming the presence of BUB1 activity in these patients. In contrast, the pH2A-T120 signal is virtually absent in P2 cells (Fig. 1H and fig. S2D). Occasionally, particularly upon colchicine treatment, some cells display residual pH2A, in contrast to HAP1-BUB1KO cells (*22*), in which no residual staining was ever observed. These results suggest that P2 cells express a truncated version that behaves very closely to a kinase-dead version. In conclusion, P2 cells express reduced levels of a protein with impaired kinase activity, whereas P1 cells express even lower levels of a full-length protein.

### BUB1 patient cells display impaired mitotic fidelity

**Fig. 2.**
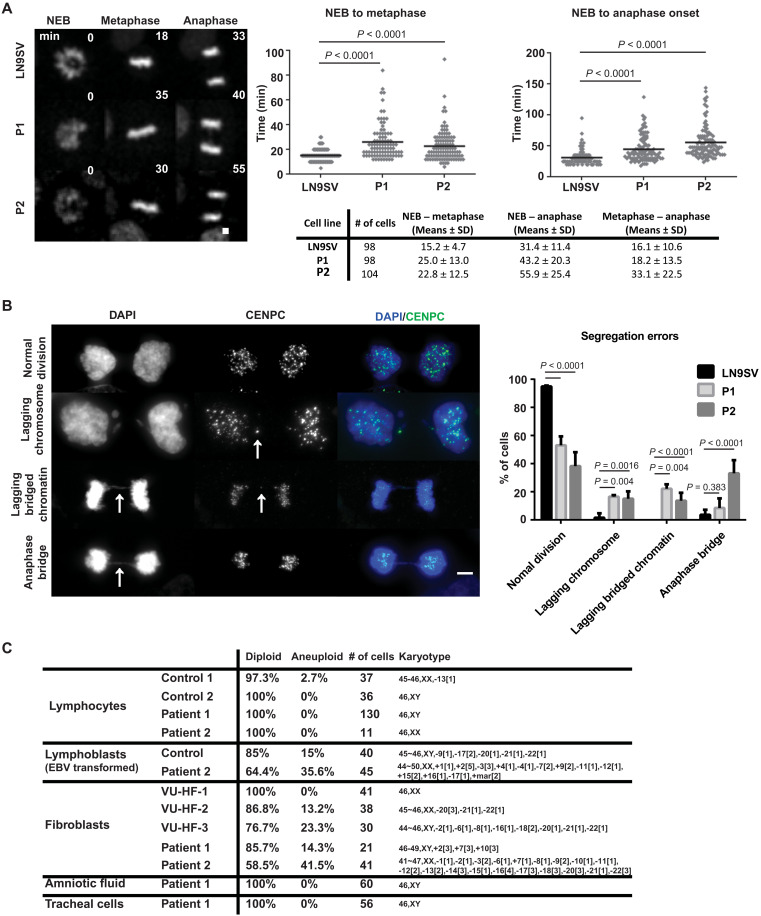
BUB1 patient cells display impaired mitotic fidelity. (**A**) Cells were filmed as they progressed through mitosis and the time from NEB until metaphase alignment (left) or anaphase onset (right) measured, as summarized in the table. Representative images are shown, with the time after NEB indicated (minutes). Cells from at least three independent experiments were analyzed. (**B**) Chromosome segregation errors were quantified in unsynchronized cells. Images depict examples of each category scored: lagging chromosomes, lagging bridged chromatin (DNA bridge with centromere staining), and anaphase bridges (defined when no centromere staining was visible within the DNA bridge). Category differences are highlighted by white arrows. A total of 65 (LN9SV), 73 (P1), or 63 (P2) late-mitosis cells were analyzed, derived from at least three independent experiments. (**C**) Cytogenetic analysis of nontransformed lymphocytes, Epstein-Barr virus (EBV)–transformed lymphoblast cell lines, primary fibroblast cell lines, amniotic fluid, and tracheal cells obtained from P1, P2, and unrelated controls. Scale bars, 5 μm. Presented *P* values were calculated using a Kruskal-Wallis test.

To investigate the functional consequences of *BUB1* mutations in patient cells, we first analyzed the kinetics of mitosis progression using live-cell imaging. Both patients exhibited significant delays, measured from NEB to metaphase (defined as the point at which all chromosomes are aligned at the metaphase plate), as well as from NEB to anaphase onset (Fig. 2A). In addition to prolonged chromosome alignment (NEB-metaphase duration) in both patients, P2 cells display a further delay to anaphase onset, suggesting compromised SAC silencing and/or APC/C activation (*44*). Analysis of live-cell imaging samples reveals that both patients’ cells display a high frequency of segregation defects at mitotic exit (36.1% in P1 and 46.0% in P2, relative to 9.3% of errors observed in controls). Next, we used high-resolution imaging of fixed cells at late anaphase/telophase to assess the types of segregation defects observed (Fig. 2B). Both patients exhibited an increased number of lagging chromosomes (whole chromatids with a centromere marker) and lagging chromosomes with DNA bridges. In contrast to P1 cells, P2 cells also display a high frequency of anaphase bridges without lagging centromeres, accounting for most of the mitotic defects observed. These differences in the observed mitotic errors suggest that distinct pathways are differentially affected in the two patients.

The high frequency of segregation defects may result in aneuploidy. Considering that heterozygous germline *BUB1* mutations were previously linked with aneuploidy in multiple tissues (*40*), we karyotyped a variety of available tissues and cell lines to assess whether aneuploidy occurs in our patients (Fig. 2C). Whereas we detected normal karyotype in all P1 investigated cell types and P2 lymphocytes, considerable chromosome number alterations were seen in P2 lymphoblasts (35.6%) and primary fibroblasts (41.5%). Since aneuploidy was hardly detected in P1 cells, it appears that in contrast to a kinase-dead BUB1 protein product, residual levels of fully functional BUB1 are sufficient to prevent aneuploidy.

### BUB1 patient cells have a functional SAC

**Fig. 3.**
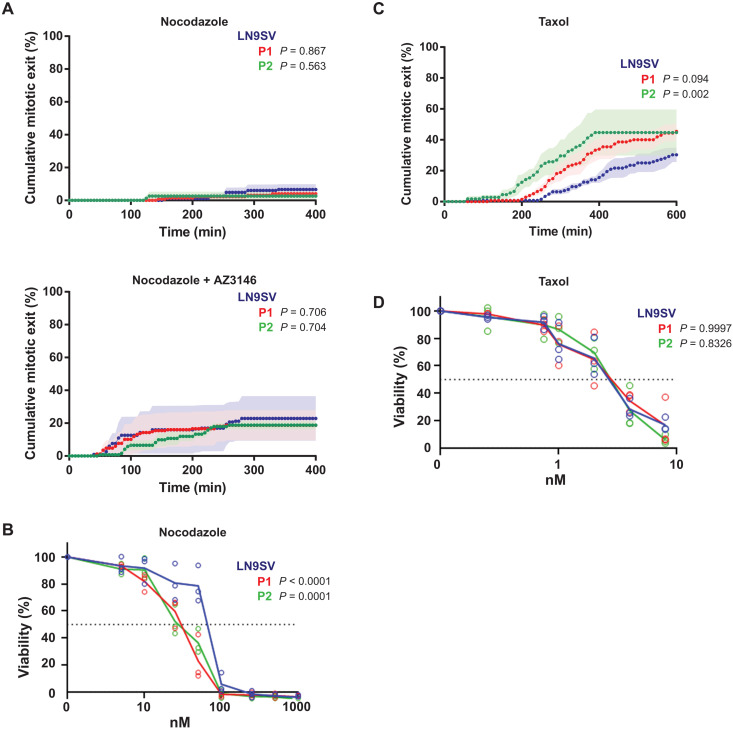
BUB1 patients’ cells have a functional SAC. (**A**) Cumulative mitotic exit of cells treated with 3.3 μM nocodazole (top; LN9SV *n* = 121, P1 *n* = 112, and P2 *n* = 65 cells) or 3.3 μM nocodazole combined with 3 μM AZ3146 (bottom; LN9SV *n* = 112, P1 *n* = 105, and P2 *n* = 107). Mitotic progression was filmed for 6 hours. Shown are means (dotted line) ± SEM (solid shading in a lighter color) from at least three independent experiments. (**B**) Cells were continuously exposed to increasing concentrations of nocodazole. After three population doublings of untreated cells, cells were counted and plotted as a percentage of untreated cells. Mean and individual data points from three independent experiments are shown. (**C**) Cumulative exit was quantified in cells treated with 20 nM Taxol and imaged for 12 hours. LN9SV *n* = 102, P1 *n* = 99, and P2 *n* = 70 cells from three experiments for each condition. Mean values are shown, and SEM is represented as solid shading in a lighter color of each corresponding condition. (**D**) Cells were continuously exposed to increasing concentrations of Taxol, and survival was assessed as in (B). Statistical analysis of the cumulative exit (A and C) was performed using Kaplan-Meier survival curves computed for each condition/drug combinations and assessed for statistical differences using the log-rank test. Differences in drug sensitivity (B and D) between patient cell lines and LN9SV were statistically assessed using beta regression.

Although BUB1 was shown to promote SAC by acting as a scaffold for MCC assembly on unattached kinetochores (*45*), our observation that mitosis duration is prolonged in patient cells (Fig. 2, A and B) suggests that these cells maintain a functional SAC that delays mitotic progression in the presence of alignment defects. To further examine SAC functionality, we evaluated the ability to maintain a mitotic arrest in a SAC-sensitized experimental layout. A comparable fraction of patient cells and control cells remained arrested in metaphase during a 6-hour nocodazole treatment with or without a low dose of monopolar spindle 1 inhibitor to sensitize SAC (Fig. 3A). These results are consistent with recent observations that very low levels of BUB1 (3 to 30%) suffice a functional SAC response (*21*, *23*, *34*, *35*) and suggest that SAC impairment is a very minor contributor, if at all, to the observed chromosome segregation errors. Nevertheless, patient cells display increased nocodazole sensitivity in growth inhibition assays (Fig. 3B), which may be caused by a cumulative effect of impairing other microtubule-dependent pathways. When challenged with Taxol, we observed that P2 cells, but not P1, show faster kinetics in mitotic exit (Fig. 3C) despite not being hypersensitive to this drug in growth inhibition assays (Fig. 3D).

Overall, these results suggest that both patients have a functional SAC. The slight difference in Taxol response in P2 cells may be more related with an impairment in error correction (see below), rather than defective SAC, as impairment in error correction response is sufficient for Taxol override (*46*, *47*).

### Distinct molecular mechanisms underlie alignment defects in BUB1 patient cells

**Fig. 4.**
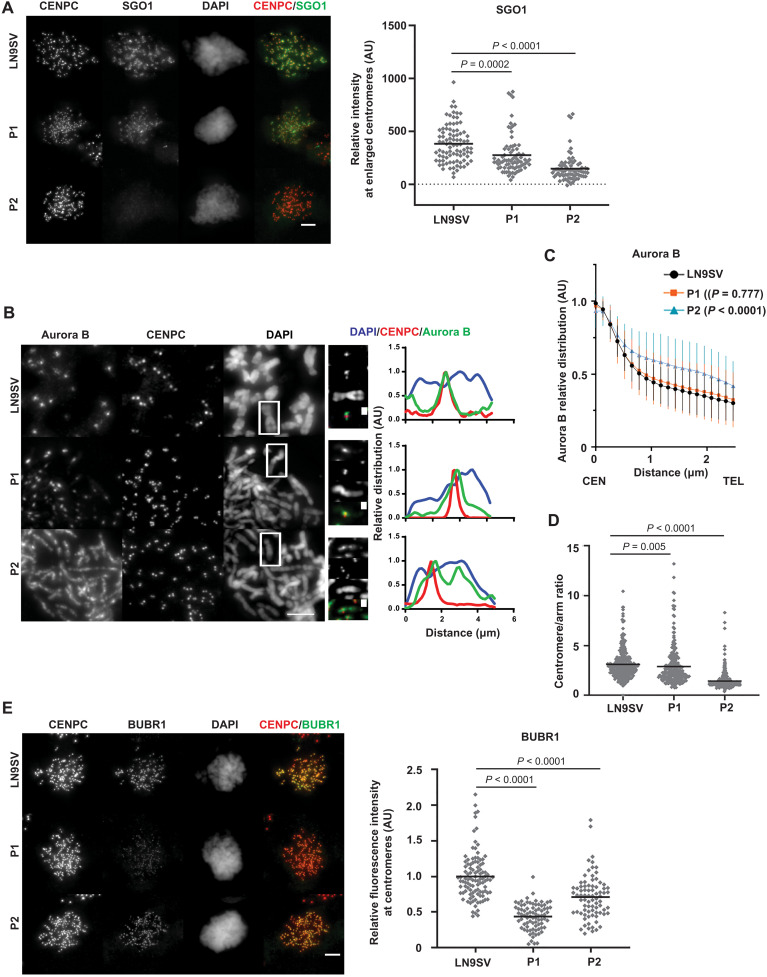
Key molecular targets of BUB1 are differentially affected in patient cells. (**A**) Colchicine-arrested cells were stained for CENPC and SGO1. Centromeric region was defined on the basis of CENPC staining, enlarged by 0.2 μm, and used to measure SGO1 mean intensity within that region. Each dot represents one cell, and black lines represent the mean per condition. LN9SV *n* = 89, P1 *n* = 79, and P2 *n* = 77 cells from three independent experiments. (**B**) Chromosomal spreads were stained for Aurora B and CENPC. Right panels depict relative chromosomal distribution of Aurora B, CENPC, and DAPI of the signaled chromosome. These plot profiles depict signal along chromosomal length, normalized to the maximum plot value within each set. (**C**) Bulk analysis of relative chromosome distribution of Aurora B along chromosomal arms (from centromere, CEN to telomere, TEL). Plot profiles were obtained as in (B), and the graph depicts the average (± SD) of approximately 100 chromosomes per condition from at least three independent experiments. *P* values refer to the comparison of relative intensities 1.5 μm away from the centromere. (**D**) Centromere/arm ratio of Aurora B signal measured from chromosomal spreads. At least 50 cells were quantified from three independent experiments. Each dot represents a chromosome. (**E**) Colchicine-arrested cells were stained for BUBR1 and CENPC. BUBR1 intensity within the CENPC-defined region was measured. LN9SV *n* = 105, P1 *n* = 80, and P2 *n* = 80 cells from three independent experiments. Each dot represents a cell, and black lines represent the mean. Each independent dataset was normalized to the average intensity of LN9SV. Scale bars, 5 μm, except on the enlarged images of the signaled chromosomes (1 μm). Presented *P* values were calculated using a Kruskal-Wallis test.

BUB1 is also involved in multiple pathways important for proper chromosome alignment. For example, BUB1-dependent H2A-T120 phosphorylation recruits SGO1, which subsequently allows docking of Aurora B, to detect and correct errors in attachment (*48*, *49*). SGO1 staining in prometaphase cells revealed a strong reduction of (peri)centromeric SGO1 in P2 cells, whereas SGO1 is only partially reduced in P1 cells (Fig. 4A). We next evaluated Aurora B centromeric localization by costaining the centromere marker CENPC on prometaphase cells, analyzed its distribution in chromosome spreads, and estimated the centromere/arm ratio (Fig. 4, B to D). Aurora B centromere/arm ratio is slightly reduced in P1 cells (Fig. 4D), but the signal is still mostly restricted to the centromeric region (Fig. 4, B and C). In P2 cells, Aurora B distribution along chromosomal length is more severely affected: Its signal is detected throughout the chromosome length (at the interchromatid region), as illustrated by the higher relative amounts observed in centromere-distal regions in intensity plot profiles along chromosome length (Fig. 4, B and C). In agreement, P2 cells show a strong reduction in the centromere/arm ratio of Aurora B distribution (Fig. 4D). This abnormal localization is not caused by a reduction in the overall levels of Aurora B in metaphase chromosomes (fig. S3A). The abnormal Aurora B distribution observed in these patients (particularly P2) is consistent with prior reports on BUB1 kinase inhibition (*50*, *51*) and could underlie the observed alignment defects in these patients (Fig. 2A).

Another contributor of chromosome alignment is BUBR1, whose kinetochore recruitment is regulated by BUB1, to ensure a dual mitotic role in both the SAC and chromosome alignment (*2*, *26*). Both patients displayed a significant decrease in BUBR1 kinetochore levels (Fig. 4E and fig. S3B). This effect was more severe in P1 cells, which lost about half of the BUBR1 pool from kinetochores, comparable to previous observations in HeLa BUB1 KO cells (*26*). These findings suggest that BUBR1 localization is more sensitive to overall BUB1 levels than to BUB1 kinase activity.

Given the importance of both Aurora B and BUBR1 in error correction for the fidelity of the mitotic cell division, we evaluated the ability to correct aberrant kinetochore-microtubule attachments induced by a transient Eg5 inhibition S-trityl-L-cysteine (STLC). Two hours after STLC washout, we observed a higher frequency of lagging chromosomes in late anaphase or telophase in patient cells, relative to unperturbed conditions, suggesting the presence of defective attachments that were not resolved at the time of exit (fig. S3C). Live imaging analysis showed a similar time from STLC release to anaphase onset among the different conditions (fig. S3D). STLC sensitivity seems to correlate with decreased BUB1 kinase activity and Aurora B recruitment (fig. S3E), suggesting that error correction is impaired. In summary, the two patients have chromosome alignment defects that may be caused by distinct molecular pathways. In P1 cells, this may be primarily attributed to defective BUBR1 localization, which is more sensitive to overall BUB1 levels than its kinase activity. P2 cells, in contrast, display absent pH2A-T120 phosphorylation and hence a higher impact on chromosomal distribution of Aurora B.

### Cohesion defects do not accelerate cohesion fatigue in BUB1 patient cells

**Fig. 5.**
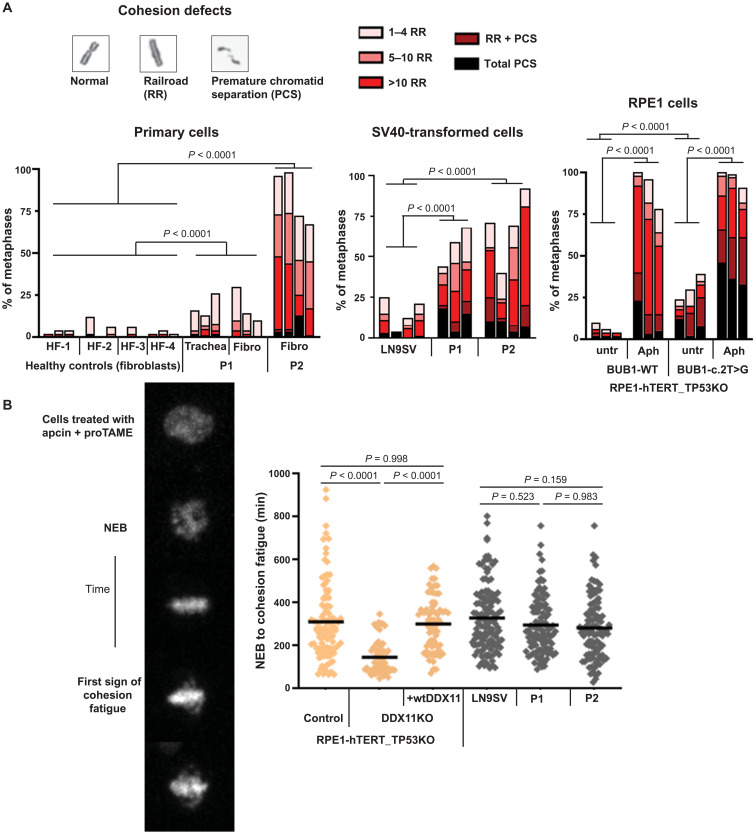
Cohesion defects do not accelerate cohesion fatigue in BUB1 patient cells. (**A**) Cohesion defect analysis of indicated cell lines. Per experiment, 50 metaphases were analyzed for each sample. Multiple independent experiments are shown as separate bars. Aph, aphidicolin (16 hours, 1 μM). *P* values were calculated using a Cochran-Mantel-Haenszel statistical test to compare the frequency of total cohesion defects per condition. (**B**) Cells were treated with 20 μM proTAME and 100 μM apcin, and the time from NEB to the moment at which single chromatids start escaping the metaphase plate (cohesion fatigue) was assessed by live-cell imaging. Each dot represents one cell. *n* ≥ 80 from at least three independent experiments. Statistical analysis was performed using one-way ANOVA.

In addition to chromosome alignment, BUB1 also contributes to the protection of centromeric cohesion from phosphorylation-dependent cohesin removal (known as the prophase pathway) via pH2A-T120–dependent SGO1 recruitment (*27*–*31*). Hence, we hypothesized that BUB1-deficient cells could also have cohesion defects. To probe for this, we used metaphase spreads of colchicine-treated cells, the classical diagnostic method to evaluate cohesion defects, to analyze sister chromatid cohesion in primary fibroblasts. In line with the observed effects on SGO1 localization, this revealed substantial cohesion defects in P2 and only a mild effect in P1 (Fig. 5A). The cohesion defects in P1 are markedly enhanced in SV40-transformed cells, likely related to SV40-induced DNA replication stress (*52*). In agreement, RPE1-*BUB1* mutant cells display mild cohesion loss, which is severely enhanced by aphidicolin-induced DNA replication stress (Fig. 5A).

Next, we assessed in vivo cohesion strength by cotreatment of cells with the APC/C inhibitors proTAME and apcin and measurement of the time from NEB until “cohesion fatigue”: asynchronous escape of sister chromatids from the metaphase plate (*53*). We validated the assay using RPE1-TP53KO/DDX11KO cells, which contain considerable cohesion defects (*54*, *55*). In these cells, the time from NEB to cohesion fatigue was much shorter when compared to control cells, and it was restored by reintroduction of WT DDX11 (Fig. 5B). However, cohesion fatigue was not accelerated in the BUB1 patient cells, indicating that sister chromatids remain functionally connected during a prolonged metaphase with intact spindle pulling forces.

### BUB1 patient cells show impaired centromeric recruitment of TOP2A

**Fig. 6.**
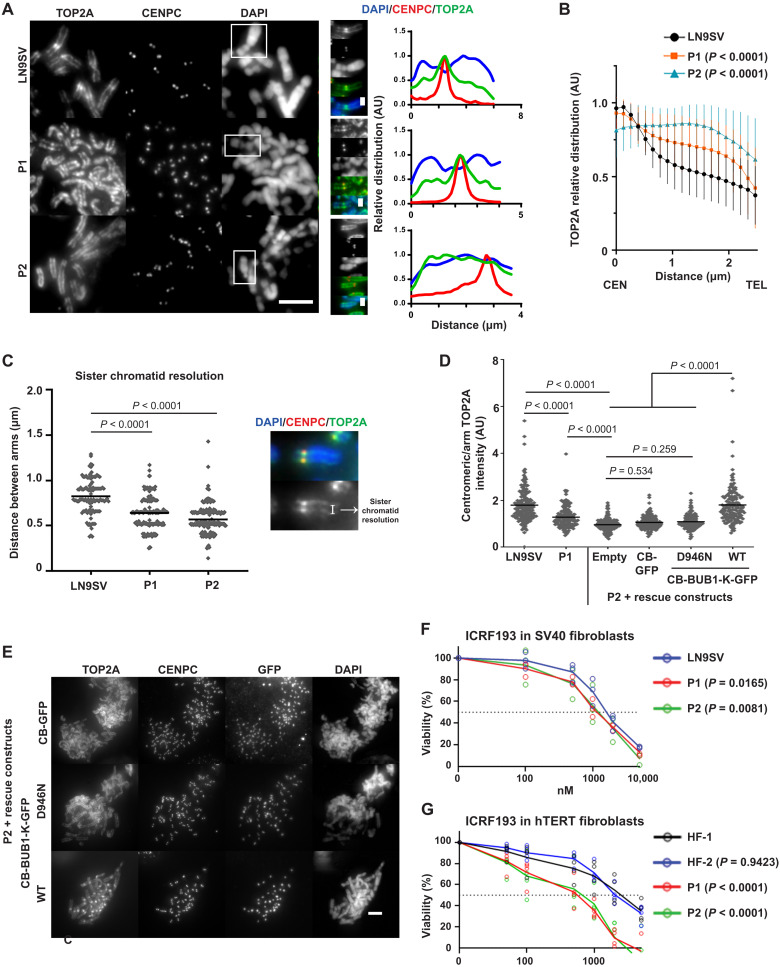
BUB1 patient cells show impaired centromeric recruitment of TOP2A. (**A**) Representative pictures of chromosomal spreads of P1, P2, and control cells stained for TOP2A and CENPC. Right panels depict relative chromosomal distribution of TOP2A/CENPC/DAPI of the signaled chromosome. These plot profiles depict signal along chromosomal length, normalized to the maximum plot value within each set. (**B**) Bulk analysis of relative chromosome distribution of TOP2A along chromosomal arms (from centromere, CEN to telomere, TEL). Plot profiles were obtained as in (A), and graph depicts average (± SD) of approximately 100 chromosomes per condition from at least three independent experiments. (**C**) Sister chromatid resolution in the indicated conditions, estimated from the distance between TOP2A peak signals within chromosomal arms. At least 100 chromosomes were measured per condition (~30 different cells), derived from three independent experiments. (**D**) Quantification of TOP2A centromere/arm ratio in chromosomal spreads, from P2 cells transfected with a centromere-targeted, GFP-tagged BUB1 kinase domain (CB-BUB1-K-GFP), a kinase-dead mutant (D946N), or an empty construct. Five chromosomes were measured per metaphase spread. At least 100 chromosomes in each condition were quantified from three independent experiments. (**E**) Representative pictures from (D). (**F** and **G**) Indicated cells were continuously exposed to increasing concentrations of ICRF193. After three population doublings of untreated cells, cells were counted and plotted as a percentage of untreated cells. Mean and individual data points from three (F) and four (G) independent experiments are shown. Scale bars, 5 μm, except on the enlarged images of the signaled chromosomes (1 μm). Presented *P* values were calculated using a Kruskal-Wallis test, except differences in drug sensitivity, which were statistically assessed using beta regression.

Considering the recently reported role of BUB1 kinase activity in the centromeric accumulation of TOP2A (*32*), it is conceivable that unresolved sister chromatid entanglements could contribute to the observed cohesion strength. We thus monitored the accumulation of TOP2A at mitotic centromeres in chromosome spreads. As predicted, while control cells display a significant accumulation of TOP2A at centromeres, the centromeric/arm ratio of TOP2A was reduced in both patients, particularly in P2 (Fig. 6, A and B). Consequently, both patients’ cells display reduced resolution of sister chromatid arms (Fig. 6C). We cannot exclude that reduced arm resolution may also be attributed to abnormal SGO1-mediated cohesion protection along the chromosome arms (*28*). However, the high frequency of anaphase bridges observed in P2 cells (Fig. 2B) argues that unresolved catenations are a major contributor of the sister chromatid resolution defects in these cells. TOP2A localization defects could be rescued by transient expression of a centromere-targeted BUB1 kinase domain construct [CB-BUB1-K-GFP (green fluorescent protein)] but not by the empty control (CB-GFP) or a kinase domain mutant version (CB-BUB1-K-D946N; Fig. 6, D and E).

These results confirm that BUB1 kinase activity promotes the centromeric localization of TOP2A. The unresolved entanglements between sister chromatids, normally resolved by TOP2A before their separation, may mimic “robust” cohesion and explain why reduced sister chromatid cohesion does not accelerate cohesion fatigue. On that basis, BUB1 patient cells could be sensitive to TOP2A inhibition. To test this prediction, we performed viability assays in the presence of ICRF193, a catalytic inhibitor of TOP2A. BUB1 mutation caused slightly reduced viability, possibly due to the cumulative effect of TOP2A mislocalization and its inhibited activity (Fig. 6F). Because SV40-dependent p53 inhibition may, in part, mask this effect, we performed our assay in hTERT immortalized fibroblasts. In these cells, both patient-derived cells exhibited increased ICRF193 sensitivity (Fig. 6G), probably due to p53-dependent growth inhibition in response to damage that follows from segregation errors.

## DISCUSSION

Here, we describe the first two patients with biallelic germline mutations in *BUB1*, which cause a previously unknown autosomal recessively inherited neurodevelopmental disorder. In line with the presumed embryonic lethality of complete loss of BUB1 activity (*36*, *39*), we find that both patients express residual BUB1 protein. Although P1 harbors a seemingly disruptive, homozygous mutation in the start codon, the expression of alternative BUB1 isoforms has been described previously (*34*). Moreover, it has been shown for other genes that the use of non-AUG start codons could still result in some residual function (*56*, *57*). We could detect a faint band in P1 and RPE1 mutant cells with a similar size as WT-BUB1, suggesting that cells express low levels of full-length BUB1 protein. Treatment of P1 cells with a BUB1 kinase inhibitor further decreased the phosphorylation of its substrate H2A-T120, which confirms residual kinase activity. By contrast, P2 cells have virtually no kinase activity, suggesting that embryonic lethality relates to a kinase-independent role of BUB1.

Because the two patients express different levels and activity of BUB1, the mechanisms by which mitotic fidelity is impaired may not be entirely the same. For instance, BUBR1 recruitment to kinetochores is most reduced in P1 cells, thus correlating with total BUB1 levels and not kinase activity. P2 cells exhibit impaired centromere recruitment of SGO1 and abnormal distribution of Aurora B and TOP2A, while in P1 cells, these proteins appear less affected. This suggests that different proteins have different thresholds for pH2A-T120–mediated recruitment. Last, we detect different degrees of sister chromatid cohesion defects. Elevated frequency of cohesion defects in P2 cells probably results from impaired localization of the cohesin protector SGO1 and Aurora B. P1 cells have a lower frequency of cohesion loss. Possibly, the observed railroad chromosomes are exacerbated by defects in chromosome architecture due to abnormal TOP2A localization (*58*–*61*). Since we observe no accelerated cohesion fatigue, we speculate that increased DNA catenation resulting from TOP2A mislocalization leads to increased mitotic cohesion strength and, at the same time, results in segregation errors.

**Fig. 7.**
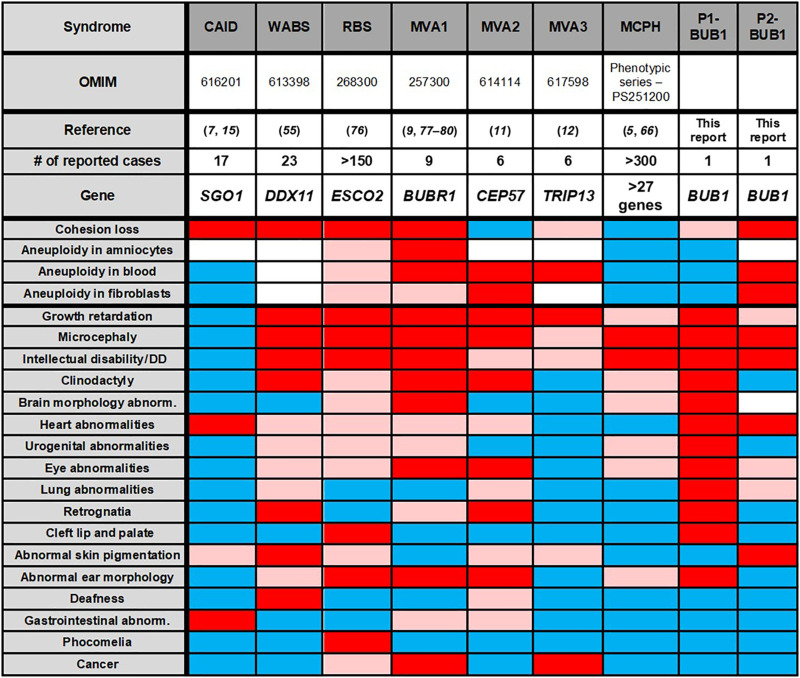
Comparison of clinical features in selected syndromes. DD, developmental delay. Red, feature present in >50% of cases; rosy, <50%; Blue, absent; white, not investigated (*76*–*80*). For description of the features of selected syndromes see references (*5*, *7*, *9*, *11*, *12*, *15*, *55*, *66*, *76*–*80*). OMIM, Online Mendelian Inheritance in Man.

The observed clinical variation between the two patients may reflect differences between kinase-dependent and kinase-independent roles of BUB1 (*62*). However, we cannot exclude that the clinical effect of *BUB1* mutations might be affected by other factors, similar to the hypomorphic mouse model *BUB1*^Δ2–3/Δ2–3^, in which viable littermates could only be generated on a mixed genetic background (*37*). Figure 7 summarizes the cellular and clinical phenotypes of the two BUB1 patients and a number of comparable syndromes.

Overlapping features include primary microcephaly, intellectual disability, varying degree of growth impairment, and a congenital heart defect [atrial septal defect II (ASDII)]. Microcephaly and intellectual disability may relate to defective mitosis (delayed mitotic timing, segregation errors, and apoptosis), leading to depletion of stem cell pools in the developing brain (*63*–*65*). Accordingly, several genes encoding for mitotic regulators have been associated with MCPH, including kinetochore proteins and regulators of chromosome organization (*5*, *66*). P2 is not dysmorphic and shows no additional congenital malformations except a small ASDII, a frequent heart abnormality found in newborns. Hence, the clinical phenotype of P2 resembles MCPH; however, aneuploidy and cohesion loss have not yet been reported in these patients (Table 1 and Fig. 7) (*5*). P1 has short stature with extreme microcephaly (occipito-frontal circumference: −7 SD), multiple congenital anomalies, and dysmorphic features (Table 1 and Fig. 7), which strongly resemble MVA syndromes and WABS.

MVA cells typically show mosaic aneuploidy and an impaired SAC (Fig. 7). In P1 cells, aneuploidy is not observed. This contrasts with a BUB1 hypomorph mouse model (which expresses <5% of BUB1) in which aneuploidy was frequently found in embryonic fibroblasts (*37*). The high frequency of aneuploidy found in this specific model system may relate with the absence of exons 2 and 3, including the highly conserved TPR domain that contributes to SAC functionality (*67*), rather than total protein levels. In P2, we observed considerable alterations in chromosome numbers in patient-derived cell lines but not in lymphocytes. This tissue-specific aneuploidy may be explained by an altered balance between the rates of missegregation and apoptosis, which may be affected by clearance of aneuploid cells by the immune system.

Moreover, we find that SAC function is largely proficient in cells from both patients. Although the hypomorphic *BUB1* mutations may have a particularly mild, context-dependent effect on SAC function and a few MVA patients have been described that also lack aneuploidy (*12*), these observations argue against *BUB1* to be a typical MVA gene.

Cohesinopathies comprise a spectrum of syndromes caused by mutations that directly affect cohesin biology. WABS, RBS, and CAID exhibit spontaneous railroad chromosomes and/or PCS (Fig. 7), which we also observe in both BUB1 patients. Cornelia de Lange syndrome (CdLS), the most frequently occurring cohesinopathy, exhibits no obvious defects in sister chromatid cohesion (*68*). The clinical symptoms of CdLS are thought to originate from deregulated gene expression [reviewed in (*69*, *70*)]. It is, at present, not clear to what extent a reduction of sister chromatid cohesion in mitosis contributes to the etiology of the different diseases. Mutations in *ESCO2* and *DDX11* both impair cohesion establishment and affect not only the integrity of mitotic cohesion but also other roles of cohesin in three-dimensional genome organization. In particular, dysregulated nucleolar organization, required for ribosome biogenesis and protein translation, has been proposed to contribute to RBS and WABS etiology (*71*, *72*).

Recently, we and others showed that partial cohesin loss to levels that do not trigger sister chromatid disjunction leads to compromised chromosome alignment and susceptibility of chromosome missegregation (*53*, *73*). These findings suggest that the contribution of mitotic failure in the context of rare syndromes, including RBS and WABS, may go beyond severe sister chromosome separation defects. In contrast to direct regulators of cohesion establishment, the function of BUB1 is restricted to mitotic processes. Thus, cohesion loss observed in these patients results from mitosis-specific failures in cohesion protection, which would not affect global chromatin organization in interphase. The clinical phenotype of BUB1 patients is much less severe as compared to that of patients with RBS and WABS. To further evaluate whether the existing clinical overlap could be attributed to a shared susceptibility to chromosome missegregation (*53*, *73*), a careful analysis of mitotic chromosome structures and CIN in the different patient cells, as well as proper in vivo disease models, is required.

Whereas patients with MVA are at risk for developing cancer (*9*, *12*), cohesinopathies and MCPH are not associated with cancer predisposition. For *BUB1*-associated disorders, this remains speculative. Transgenic Bub1 mouse models suggest that increased tumorigenesis can result from low levels (<5% of total protein) (*37*) but not from kinase deficiency (*38*). Thus far, both BUB1 patients show no signs of tumor development. Long-term follow-up and characterization of more patients are necessary to clarify a potentially enhanced cancer risk and to fully describe the phenotypic spectrum associated with BUB1-related neurodevelopmental disorders.

## MATERIALS AND METHODS

### Sequencing

WES from DNA from blood and RNA sequencing from primary fibroblasts were performed in P1 as described previously (*74*). For WES of P2 and parents, genomic DNA was isolated from blood, and 2.5 μg was sheared on a Covaris S2 instrument (Covaris, Woburn, MA). DNA libraries were prepared using Kapa Biosystems reagents (Kapa Biosystems, Wilmington, MA), and 1.0 μg of library was used for enrichment with Roche/NimbleGen SeqCap EZ MedExome (Roche, Basel, Switzerland) according to the manufacturer’s protocol. Sequencing was performed on an Illumina HiSeq 2500 platform (Illumina, San Diego, CA, USA) with 125–base pair paired-end reads. More than 88% of the capture region was covered ≥30× with a mean bait coverage of at least 90× for each sample. Variant calling was performed using an in-house analysis pipeline. Alignment of sequence reads to the human genome (hg19) was performed with the Burrows-Wheeler Aligner tool (BWA-MEM v0.7.10) using default settings. Subsequently, Picard Tools (v1.111; http://picard.sourceforge.net/) was used for sorting and marking duplicates. For local realignment and base quality score recalibration, we used the Genome Analysis Toolkit (GATK; v3.3-0; Broad Institute, Cambridge, MA), and for variant calling, we used the GATK HaplotypeCaller. Variants were filter-tagged using the GATK VariantFiltration and annotated by snpEff (v4.0). Variant prioritization was performed using Cartagenia Bench Lab NGS (Agilent Technologies, Santa Clara, USA). For Sanger sequencing, genomic DNA was isolated with the blood and cell culture DNA maxi kit (Qiagen) and RNA with the high pure isolation kit (Roche), followed by cDNA synthesis using the iScript (Bio-Rad). Primer sequences are provided in table S1.

### Cell lines and tissue culture

Primary human cells were cultivated from skin biopsies of P1 and P2 as well as from a surgery sample from the trachea of P1. Fibroblast cells were transformed with SV40 large T antigen. SV40-transformed P2 cells were transfected, using Lipofectamine LTX reagent for 48 hours according to the manufacturer’s protocol, with CB-GFP, CB-BUB1-K-GFP, or CB-BUB1-K-D946N-GFP, provided by F. Wang (*32*); RPE1-TP53KO, RPE1-TP53KO-DDX11KO, and RPE1-TP53KO-DDX11KO + wtDDX11 cells (*52*); SV40-transformed control fibroblast cell line LN9SV (male) (*75*); and primary and SV40-transformed fibroblasts from healthy adult donors HF-1 (female), HF-2 (female), HF-3 (male), and HF-4 (male) (*52*) were described previously. HAP1 WT and HAP1-BUB1KO cells (*22*) were described previously. All cell lines were cultured in Dulbecco’s modified Eagle’s medium (BioWest); supplemented with 10% fetal calf serum (Sigma-Aldrich), 1 mM sodium pyruvate, and 100 U of penicillin/streptomycin (Gibco); and maintained at 37°C and 5% CO_2_.

### Quantitative reverse transcription polymerase chain reaction

Quantitative reverse transcription polymerase chain reaction (qRT-PCR) was performed using SYBR Green (Roche) on a LightCycler 480 (Roche). Levels were normalized to the geometric mean of two housekeeping genes (hypoxanthine-guanine phosphoribosyltransferase and TATA box–binding protein). Primer sequences are provided in table S1.

### Immunoblotting

Cells were lysed in lysis buffer [50 mM tris-HCl (pH 7.4), 150 mM NaCl, and 1% Triton X-100] with protease and phosphatase inhibitors (Roche). Proteins were separated by 4 to 15% SDS–polyacrylamide gel electrophoresis (Bio-Rad) and transferred to Immobilon-P membranes (Millipore). Membranes were blocked in 5% dry milk in TBST-T [10 mM tris-HCl (pH 7.4), 150 mM NaCl, and 0.04% Tween 20] and incubated with primary and peroxidase-conjugated secondary antibodies (1:10,000; DAKO, Glostrup, Denmark), and bands were visualized by chemiluminescence (Amersham). Antibodies used for detection are rabbit anti-BUB1 (1:1000; A300-373A, Bethyl and ab9000, Abcam), sheep anti-BUB1 (1:500; SB1.3, gift from S. Taylor), mouse anti–α-tubulin (1:2000; B-5-1-2, #sc-23948, Santa Cruz Biotechnology), and mouse anti-CDC6 (1:500; #sc-9964, Santa Cruz Biotechnology).

### Immunofluorescence

Cells were seeded on 18-mm-diameter #1.5 coverslips 1 to 2 days before the experiment. On the day of the experiment, before fixation, cells were treated for 2 hours with 10 μM colchicine (Sigma-Aldrich), except for analysis of unsynchronized cells. For BUB1 kinase inhibition, 4 μM BAY1816032 (MedChemExpress) was used for 1 hour before fixation. Cells were fixed at room temperature (RT) with 4% formaldehyde and 1% Triton X-100 in phosphate-buffered saline (PBS) for 20 min and stained for BUB1, BUBR1, and Aurora B. For pH2A-T120 and mitotic error analysis, cells were fixed at RT with 4% formaldehyde in PBS for 5 min, followed by 5 min of permeabilization with 0.5% Triton X-100, and then fixed again. Independently of the fixation protocol, coverslips were incubated after for 5 min in PBS plus 0.1% Triton X-100 (PBS-Wash), tris-buffered saline (TBS) plus 0.1% Triton X-100, and again in PBS-Wash. For blocking and antibody dilution, we used TBS, 0.1% Triton X-100, 2% bovine serum albumin (BSA), and 0.1% sodium azide. Coverslips were blocked for 30 min at RT, and primary antibody incubations were done overnight at 4°C for BUB1 (1:100; A300-373A, Bethyl Laboratories), BUBR1 (1:100; A300-386A, Bethyl Laboratories), Aurora B (1:100; AIM-1, BD Biosciences), SGO1 (1:500; ab58023, Abcam), CENPC (1:500; PD030-MBL), and tubulin (1:200; sc-32293) and 1 hour at RT for H2ApT120 (1:1000; Active Motif) and sheep anti-BUB1 (1:1000; SB1.3, gift from S. Taylor). Secondary antibody incubations were done at RT for 1 to 2 hours (1:500; Jackson ImmunoResearch and Invitrogen). Excess antibody was removed after primary and secondary antibody incubations by three washes with PBS-Wash for 5 min. Single coverslips were mounted in Vectashield mounting medium.

### Chromosome spreads

Mitotic cells were collected by mitotic shake-off of cells pretreated with 10 μM colchicine for 30 min then resuspended in hypotonic solution (75 mM KCl) in a 1:1 ratio with cell media for 7 min at RT. Incubation period was terminated by adding 2 volumes of 2% BSA PBS 1×. Cells were spread into coverslips treated with poly-l-lysine (Sigma-Aldrich) and centrifuged at 800*g* for 4 min in a Cytospin (Thermo Fisher Scientific). After air-drying the slides for 1 min, cells were fixed using 4% formaldehyde and 1% Triton X-100 in 1× PBS for 10 min, and the immunofluorescence continued as described above, as well as its acquisition. Primary antibodies were incubated for 1 hour at RT in a humidified chamber. Aurora B (1:100; AIM-1, BD Biosciences) and TOP2A (1:1000; M042-3, MBL) were used. CENPC was used at 1:1000.

### Imaging

Images of prometaphase-arrested cells (with the exception of pH2A-T120 and BUB1) were acquired on a Zeiss Imager Z2/ApoTome.2 system, equipped with an Axiocam 105 color camera, using a 100× 1.4 numerical aperture (NA) oil immersion objective, 1.6× Optovar, 4′,6-diamidino-2-phenylindole (DAPI) + CY5 fluorescence filter sets, and 1 × 1 binning. Serial sections were acquired every 0.2 μm. All other samples (pH2A-T120, BUB1, chromosomes spreads, and mitotic errors) were acquired on a DeltaVision Core with a 100×/1.40 NA UPlan SAPO oil objective using softWoRx software (Applied Precision). Images were acquired at 1 × 1 binning using an electron multiplying charge-coupled device (EMCCD) camera and DAPI + CY5 fluorescence filter sets.

On average, 30 images per condition in three independent replicates were acquired using the same acquisition settings. Displayed images are maximum-intensity projections after background subtraction.

For time-lapse imaging, cells were seeded at optimized density (control cells, 1.7 × 10^5^ and P1 and P2 cells, 20% more) in μ-Slide 8 Wells (ibidi). The next day, fresh media containing SiR-DNA (1:2000; Spirochrome) was added 1 hour before the start of imaging. For mitotic progression, before imaging, fresh media was added to remove SiR-DNA. Cells were imaged every 3 min for 16 hours. In case of SAC analysis, cells were treated either with Taxol (20 nM; Invitrogen) or nocodazole (3.3 μM; Sigma-Aldrich) with or without AZ3146 (3 μM; Sigma-Aldrich) before imaging. Time course was done in media containing drugs (except SiR-DNA), added immediately before imaging, every 5 min for 6 hours or 10 min for 12 hours, respectively. Imaging was done at the Roper Nikon microscope (Photometrics 512 EMCCD) with full temperature atmosphere control (37°C + humidity + 5% CO_2_), using 20× objective sections with a Z-optical spacing of 1.5 μm. Cohesion strength was inferred by live-cell time-lapse imaging adapting the protocol described in (*53*). Unsynchronized cells were treated with SiR-DNA for 1 hour before imaging. Just before imaging, medium was replaced with fresh media containing 20 μM proTAME (R&D Systems) and 100 μM apcin (Sigma-Aldrich). Cells were imaged every 3 min on the Roper Nikon microscope (Photometrics 512 EMCCD) with full temperature atmosphere control (37°C + humidity + 5% CO_2_) for 16 hours, using 20× objective sections with a Z-optical spacing of 1.5 μm.

### Karyotyping

Confluent cells (70 to 80%) were treated with KaryoMAX Colcemid (0.035 μg/ml; Thermo Fisher Scientific) followed by a 4- to 16-hour incubation at 37°C (depending on the proliferation rate of cell lines). Cells were harvested, resuspended with prewarmed hypotonic solution (0.075 M KCl; Merck) for 20 min at 37°C, and washed three times with freshly prepared methanol/acetic acid fixative (3:1; VWR) on ice. Fixed cells were dropped on humid slides, treated with SSC solution (Merck) for 5 hours at 60°C, and stained with 4% Giemsa staining solution (VWR) for 6 min. Slides containing metaphases were analyzed using the scanning program Metafer4 (Metasystems). Metaphases were counted and karyotyped by using the Ikaros program (Metasystems).

### Cohesion defect analysis

Cells were incubated with demecolcin (200 ng/ml; Sigma-Aldrich) for 20 min, harvested, resuspended in 0.075 M KCl for 20 min, and fixed in methanol/acetic acid (3:1). Cells were washed in fixative three times, dropped onto glass slides, and stained with 5% Giemsa (Merck). Cohesion defects were counted in 50 metaphases per condition on two different coded slides.

### Quantitative imaging analysis

The time from NEB to metaphase and anaphase was manually assessed in coded samples. For SAC analysis, only cells that entered mitosis in the first 1 hour and 30 min of imaging were quantified. Cohesion fatigue timing was determined manually from NEB until the first signs of cohesion fatigue, defined as the first time point at which loose chromatids were observed on both sides of the arrested metaphase plate, using coded samples.

For quantitative analysis of protein levels, we used prometaphase-arrested cells (10 μM colchicine for 2 hours) and an in-house–developed macro in FIJI. Images were background subtracted and automatically segmented on the basis of DAPI (default threshold) or CENPC (MaxEntropy threshold) staining to select for the DNA or centromeric regions, respectively. Mean fluorescence intensity of the protein of interest was measured within each region of interest (ROI) (DNA, the entire DAPI-defined region), centromeres (CENPC-defined), or enlarged centromeres (CENPC-defined ROI enlarged by 0.2 μm, to enclose the inner centromere). Each value was subtracted by the mean fluorescence intensity of the cytoplasm, measured using a ~20-μm^2^ region randomly placed in the vicinity of DNA.

To obtain plot profiles describing protein distribution along chromosomal length, chromosomes were randomly selected on the basis of DNA staining to select isolated chromosomes, and a 10- to 15-pixel-wide line was drawn over each chromosome. Their intensities were obtained using plot profile (FIJI), normalized to the highest point in the dataset, and relative protein amounts were plotted with Prism. For bulk analysis of the protein distribution in different chromosomes, a similar approach was used measuring solely half of chromosome arms (starting at centromeres). Chromosomes with approximately 2.5 μm of distance from the centromere to the end were used for this average analysis to discard length variability.

For relative amounts of protein distribution (centromere/arm ratio), we used chromosome spreads, as they enable a more spatially resolved analysis of subchromosomal protein distribution. For this, mean fluorescence intensities of each protein (Aurora B/TOP2A) were measured on FIJI using a circle of 10-pixel diameter that was placed around the centromere (CenpC-defined) and chromosome arms (DAPI-defined) of the same chromosome. Approximately five isolated chromosomes were measured per spread after random selection (based on DAPI staining).

For analysis of sister chromatid resolution, we used chromosome spreads stained with TOP2A. A 4-pixel line was placed along the chromosomal width, placed close to the telomere. Sister chromatid resolution was defined by the distance between the two peaks of the TOP2A staining. Approximately five isolated chromosomes were measured per spread after random selection (based on DAPI staining).

### Statistical analysis

Comparative analysis of protein intensities/distributions/distances was performed using Prism 8 (GraphPad), using one-way analysis of variance (ANOVA) or the nonparametric Kruskal-Wallis test, depending on whether all datasets passed the D’Agostino and Pearson normality test. Kruskal-Wallis test was followed by Dunn’s multiple comparison test. To compare drug sensitivities between cell lines, we applied beta regression using R software. Post hoc comparisons between cell lines were done and corrected for multiplicity with Tukey’s test (α = 0.05). Mitotic exit times were analyzed using a survival analysis methodology, including cells for which the time of mitosis initiation was recorded but did not exit during the time lapse. Kaplan-Meier survival curves were computed for each condition/drug combinations and assessed for statistical differences using the log-rank test. *P* values were corrected for multiple testing where appropriate using Bonferroni corrections. The analysis was performed using python and the packages pandas v1.2.3, numpy v1.18.1, and lifelines v0.26.0. Statistical analysis of cohesion defects was performed using Cochran-Mantel-Haenszel test, using python statsmodels.

## Notes

View/request a protocol for this paper from *Bio-protocol*.
