# Supplementary material for: Biallelic BUB1 mutations cause microcephaly, developmental delay, and variable effects on cohesion and chromosome segregation
Source: Sci Adv. 2022 Jan 19;8(3):eabk0114. doi: 10.1126/sciadv.abk0114 (PMC8769543; doi:10.1126/sciadv.abk0114)
Supplement: Supplementary file 1 — Figs. S1 to S3 Table S1 [file sciadv.abk0114_sm.pdf]

Science Advances
AAAS

Supplementary Materials for

Biallelic *BUB1* mutations cause microcephaly, developmental delay, and variable effects on cohesion and chromosome segregation

Sara Carvalhal, Ingrid Bader, Martin A. Rooimans, Anneke B. Oostra, Jesper A. Balk, René G. Feichtinger, Christine Beichler, Michael R. Speicher, Johanna M. van Hagen, Quinten Waisfisz, Mieke van Haelst, Martijn Bruijn, Alexandra Tavares, Johannes A. Mayr, Rob M. F. Wolthuis, Raquel A. Oliveira*, Job de Lange*

*Corresponding author. Email: rcoliveira@igc.gulbenkian.pt (R.A.O.); j.delange1@amsterdamumc.nl (J.d.L.)

Published 19 January 2022, *Sci. Adv.* **8**, eabk0114 (2022)
DOI: 10.1126/sciadv.abk0114

This PDF file includes:

Figs. S1 to S3
Table S1

Figure S1

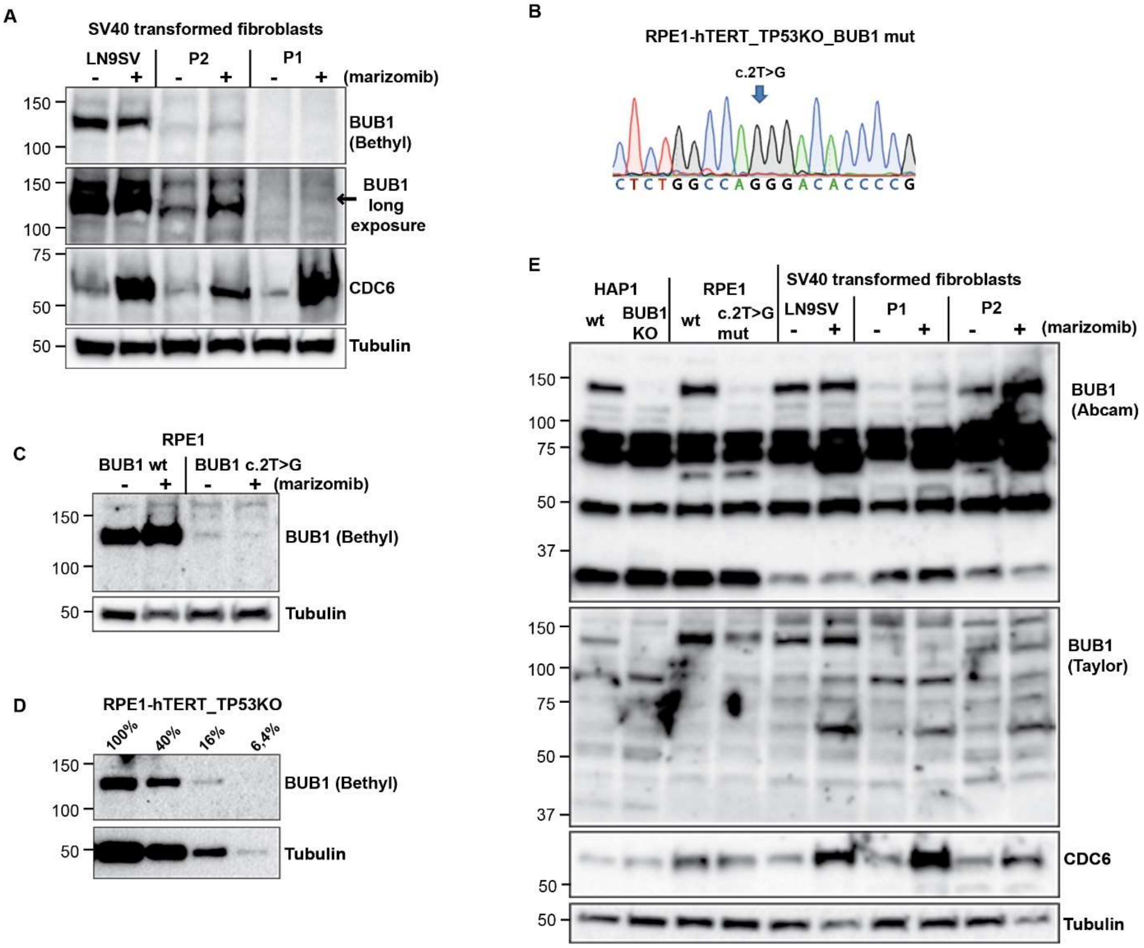

Figure S1: Residual BUB1 protein levels in patient cells.

(**A**) Western blot of SV40 transformed fibroblasts, treated with the proteasome inhibitor marizomib (6h, 500 nM). For BUB1 staining, we used Bethyl A300-373A, raised against a.a. 1-50. The arrow indicates full-length BUB1 in P1 cells. CDC6 is used as control for marizomib induced protein stabilization. (**B**) Validation of CRISPR engineered c.2T>G mutation in both BUB1 alleles in RPE1-hTERT_TP53KO cells by Sanger sequencing. (**C**) RPE1-wt and BUB1 mutant cells were treated with marizomib (6h, 500 nM) and assessed by western blot. (**D**) Untreated RPE1 control lysate was serially diluted and BUB1 levels were assessed by western blot. (**E**) Western blot of indicated cell lines; fibroblasts were treated as in (**A**). For BUB1 staining, we used an antibody raised against a.a. 1-350 (ab9000, upper blot) and an antibody raised against a.a. 336-489 (kindly provided by Stephen Taylor, lower blot). Note that no shorter band is detected specifically in RPE1-mut c.2T>G or in P1 cells. CDC6 was used as control for marizomib induced protein stabilization.

Figure S2

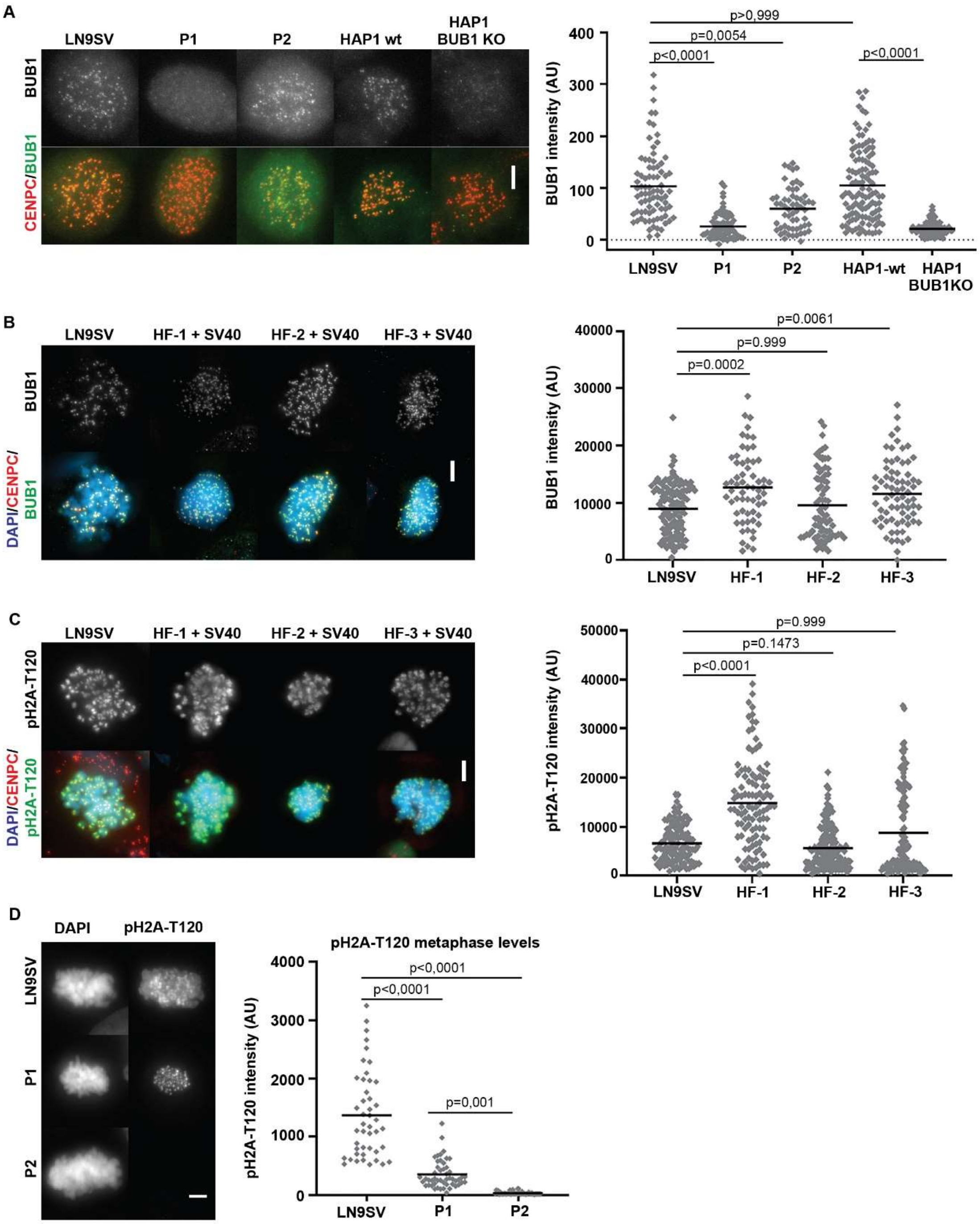
A
LN9SV
P1
P2
HAP1 wt
HAP1 BUB1 KO
BUB1
CENPC/BUB1
p>0,999
p=0,0054
p<0,0001
p<0,0001
BUB1 intensity (AU)
400
300
200
100
0
LN9SV
P1
P2
HAP1-wt
HAP1 BUB1KO
B
LN9SV
HF-1 + SV40
HF-2 + SV40
HF-3 + SV40
BUB1
DAPI/CENPC/BUB1
p=0.0061
p=0.999
p=0.0002
BUB1 intensity (AU)
40000
30000
20000
10000
0
LN9SV
HF-1
HF-2
HF-3
C
LN9SV
HF-1 + SV40
HF-2 + SV40
HF-3 + SV40
pH2A-T120
DAPI/CENPC/pH2A-T120
p=0.999
p=0.1473
p<0.0001
pH2A-T120 intensity (AU)
50000
40000
30000
20000
10000
0
LN9SV
HF-1
HF-2
HF-3
D
DAPI
pH2A-T120
LN9SV
P1
P2
pH2A-T120 metaphase levels
p<0,0001
p<0,0001
p=0,001
pH2A-T120 intensity (AU)
4000
3000
2000
1000
0
LN9SV
P1
P2

Figure S2: Reduced levels of BUB1 in P1 cells and impaired BUB1 kinase activity in P2 cells. (**A**) Indicated cell lines were arrested with colchicine and centromeric BUB1 signal was measured by staining with an antibody raised against a.a. 336-489 (kindly provided by Stephen Taylor). Each dot represents relative intensity from one cell. LN9SV (n=81), P1 (n=74), P2 (n=58), HAP-1 wt (n= 114), HAP-1 BUB1 KO (n=107) cells from three independent experiments. (**B**) LN9SV SV40 transformed fibroblasts were compared with three other SV40 transformed fibroblasts from healthy donors. Colchicine arrested cells were stained for CENPC and BUB1. BUB1 relative intensity was measured at centromeric region (defined by CENPC staining). Each dot represents one cells, results obtained from 3 independent experiments (LN9SV= 100, HF-1= 41, HF-2=84 and HF-3= 73 cells). (**C**) Cells were arrested in colchicine and stained for pH2A-T120 (right panel), CENPC and DNA. Relative intensity of pH2A-T120 was measured within the DNA region in SV40 transformed fibroblasts from healthy donors. Each dot represents the relative intensity obtained in one cell. Approximately 100 cells were measured from three independent experiments. Note that LN9SV behaves comparable to other controls. (**D**) Metaphase plates from unsynchronized cells were stained for pH2A-T120 and their relative intensity was measured on the DNA region. N=50 cells from three independent experiments.

Figure S3

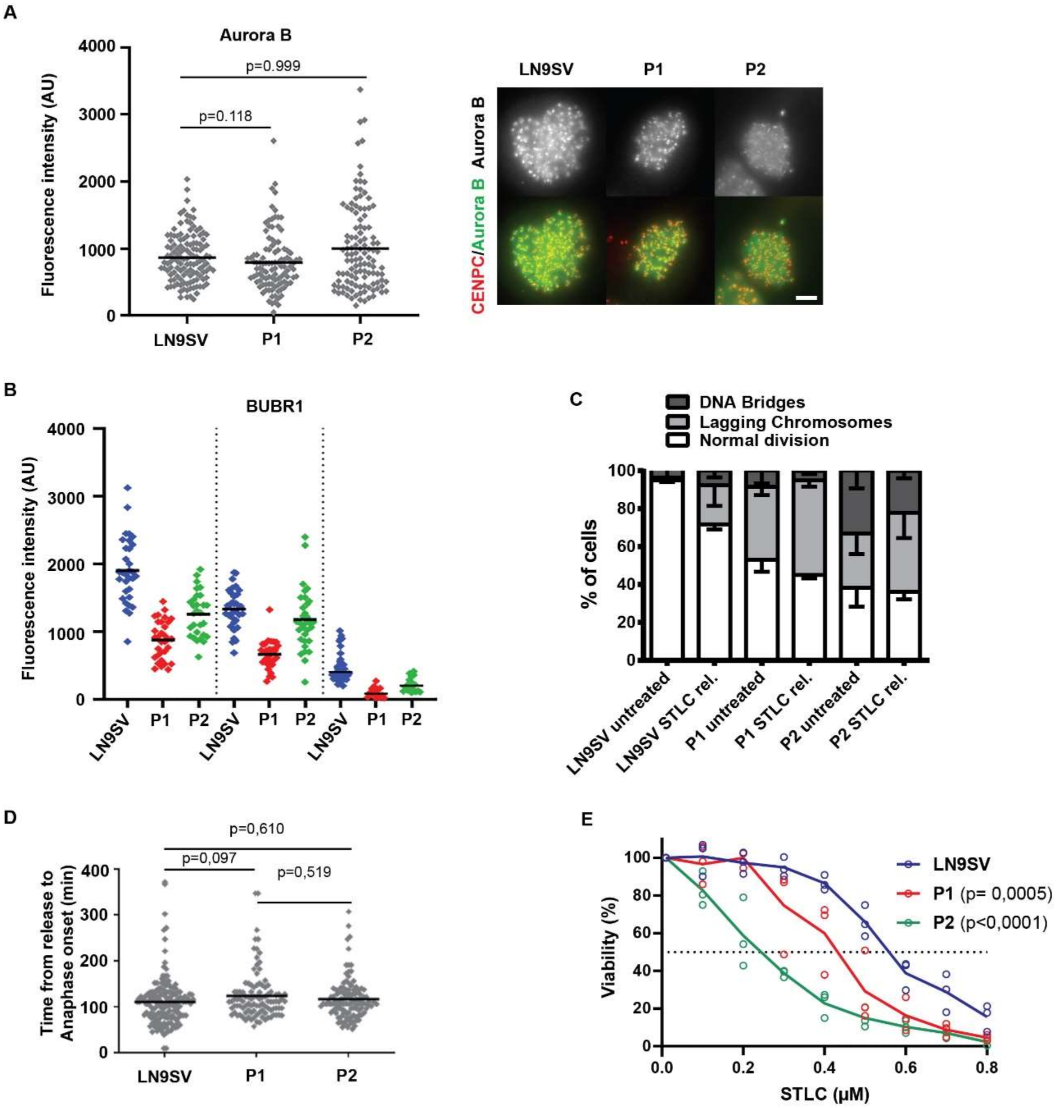
A
Aurora B
Fluorescence intensity (AU)
4000
3000
2000
1000
0
p=0.999
p=0.118
LN9SV
P1
P2
LN9SV
P1
P2
Aurora B
CENPC/Aurora B
B
BUBR1
Fluorescence intensity (AU)
4000
3000
2000
1000
0
LN9SV
P1
P2
LN9SV
P1
P2
LN9SV
P1
P2
C
DNA Bridges
Lagging Chromosomes
Normal division
% of cells
100
80
60
40
20
0
LN9SV untreated
LN9SV STLC rel.
P1 untreated
P1 STLC rel.
P2 untreated
P2 STLC rel.
D
Time from release to Anaphase onset (min)
400
300
200
100
0
p=0,610
p=0,097
p=0,519
LN9SV
P1
P2
E
Viability (%)
100
80
60
40
20
0
0.0
0.2
0.4
0.6
0.8
STLC (μM)
LN9SV
P1 (p= 0,0005)
P2 (p<0,0001)

Figure S3: No reduced Aurora B intensity in patient cells. (**A**) Colchicine arrested cells were stained for Aurora B and their overall relative intensity was measured (defined by the DNA region). Each dot represents a measurement from one cell. At least 100 cells were measured at each condition from four independent experiments. (**B**) BUBR1 raw intensities measured within the CENPC region were obtained for each independent experiment performed in colchicine-arrested cells stained for BUBR1, CENPC and DAPI. Each independent data set was normalized to the average intensity of LN9SV to devoid extrinsic fluorescence variability as represented in Figure 4E. (**C**) Cells were treated S-trityl-L-cysteine (STLC, 2.5 μM for 3 hours). Two hours after wash-out, chromosome segregation errors were evaluated in late anaphase or telophase. Lagging chromosomes also include lagging chromosomes associated with chromatin bridges. n≥50 cells at each condition from three independent experiments. (**D**) Time from STLC wash-out until anaphase. n≥100 cells at each condition from three independent experiments. STLC treatment performed as in (C). (**E**) Cells were continuously exposed to increasing concentrations of STLC. After three population doublings of untreated cells, cells were counted and plotted as percentage of untreated cells. Mean and individual data points from three independent experiments are shown. Scale bar = 5 μm. Differences in drug sensitivity between patient cell lines and LN9SV were statistically assessed using beta regression. All other p-values were calculated using a Kruskal–Wallis test.

Table S1: Primer sequences

Primer name	Sequence
**Sanger genomic DNA BUB1 start Fw**	CTGCGCGGGGTATTCGAATC
**Sanger genomic DNA BUB1 start Rv**	GAGCTCTGAGGGCCTAACGAAT
**Sanger genomic DNA BUB1 dupG Fw**	TTACCTGTGAGGCAGAGTTGGG
**Sanger genomic DNA BUB1 dupG Rv**	CGCTAAGCACATGCAGAACACT
**Sanger genomic DNA BUB1 splice Fw**	TTTGCCCAGGTGTACGAAGCTA
**Sanger genomic DNA BUB1 splice Fw**	AAATTGAGAGCTTTGCCCCTGC
**Sanger cDNA exon 18 Fw**	TGTACCCAGGCGACTTTGGATT
**Sanger cDNA exon 24 Rv**	TGCAGCAACCCCAAAGTAATCG
**qRT-PCR BUB1 exon 1-2 Fw**	GTTTGCGGTTCAGGTTTGG
**qRT-PCR BUB1 exon 1-2 Rv**	TCATTGCCCTTGTAGCTCTG
**qRT-PCR BUB1 exon 10-11 Fw**	TTGCTCCTCCTGTTCCTTTG
**qRT-PCR BUB1 exon 10-11 Rv**	CTTTGATCTCTGCTCCACTCTG
**qRT-PCR BUB1 exon 24-25 Fw**	AAATGAAGGAGGAGAGTGTAAGC
**qRT-PCR BUB1 exon 24-25 Rv**	TGGAAGATGATGACAATCTGGAATA
**qRT-PCR HPRT Fw**	TGACACTGGGAAAACAATGCA
**qRT-PCR HPRT Rv**	GGTCCTTTTCACCAGCAAGCT
**qRT-PCR TBP Fw**	TGCACAGGAGCCAAGAGTGAA
**qRT-PCR TBP Rv**	CACATCACAGCTCCCCACCA
